# Growth of human gastric cancer cells in nude mice is delayed by a ketogenic diet supplemented with omega-3 fatty acids and medium-chain triglycerides

**DOI:** 10.1186/1471-2407-8-122

**Published:** 2008-04-30

**Authors:** Christoph Otto, Ulrike Kaemmerer, Bertram Illert, Bettina Muehling, Nadja Pfetzer, Rainer Wittig, Hans Ullrich Voelker, Arnulf Thiede, Johannes F Coy

**Affiliations:** 1 Experimental Transplantation Immunology, Department of Surgery, University of Würzburg Hospital, Oberdürrbacher Str. 6, D-97080 Würzburg, Germany; 2 Department of Obstetrics and Gynaecology, University of Würzburg Hospital, Josef-Schneider-Str. 4, D-97080 Würzburg, Germany; 3 Department of Surgery, University of Würzburg Hospital, Oberdürrbacher Str. 6, D-97080 Würzburg, Germany; 4 Tavartis GmbH, Kroetengasse 10, D-64853 Otzberg, Germany; 5 Institute of Pathology, University of Würzburg, Josef-Schneider-Str. 2, D-97080 Würzburg, Germany

## Abstract

**Background:**

Among the most prominent metabolic alterations in cancer cells are the increase in glucose consumption and the conversion of glucose to lactic acid via the reduction of pyruvate even in the presence of oxygen. This phenomenon, known as aerobic glycolysis or the Warburg effect, may provide a rationale for therapeutic strategies that inhibit tumour growth by administration of a ketogenic diet with average protein but low in carbohydrates and high in fat enriched with omega-3 fatty acids and medium-chain triglycerides (MCT).

**Methods:**

Twenty-four female NMRI nude mice were injected subcutaneously with tumour cells of the gastric adenocarcinoma cell line 23132/87. The animals were then randomly split into two feeding groups and fed either a ketogenic diet (KD group; n = 12) or a standard diet (SD group; n = 12) *ad libitum*. Experiments were ended upon attainment of the target tumor volume of 600 mm^3 ^to 700 mm^3^. The two diets were compared based on tumour growth and survival time (interval between tumour cell injection and attainment of target tumour volume).

**Results:**

The ketogenic diet was well accepted by the KD mice. The tumour growth in the KD group was significantly delayed compared to that in the SD group. Tumours in the KD group reached the target tumour volume at 34.2 ± 8.5 days versus only 23.3 ± 3.9 days in the SD group. After day 20, tumours in the KD group grew faster although the differences in mean tumour growth continued significantly. Importantly, they revealed significantly larger necrotic areas than tumours of the SD group and the areas with vital tumour cells appear to have had fewer vessels than tumours of the SD group. Viable tumour cells in the border zone surrounding the necrotic areas of tumours of both groups exhibited a glycolytic phenotype with expression of glucose transporter-1 and transketolase-like 1 enzyme.

**Conclusion:**

Application of an unrestricted ketogenic diet enriched with omega-3 fatty acids and MCT delayed tumour growth in a mouse xenograft model. Further studies are needed to address the impact of this diet on other tumour-relevant functions such as invasive growth and metastasis.

## Background

Cancer is caused by multiple complex processes influencing cellular proliferation, differentiation and death. Genetic alterations favouring growth drive the transformation of normal cells into malignant cells [1]. The relationship between cancer-causing genes and cellular energy metabolism is only partially understood [2]. Several authors have shown that genetic alterations promoting tumour development directly affect glucose-mediated energy metabolism [3,4]. Thompson and colleagues, for example, determined that the activated serine/threonine kinase Akt promotes glucose consumption in transformed cells without affecting the rate of oxidative phorphorylation [5].

The conversion of glucose to lactic acid via the reduction of pyruvate, even in the presence of oxygen, is known as aerobic glycolysis or the Warburg effect. Aggressive tumours frequently exhibit this metabolic alteration and reveal an increasing dependency on the glycolytic pathway for ATP generation. Most cells of non-malignant tissues, in contrast, use pyruvate to produce ATP via mitochondrial respiration in the presence of oxygen. Warburg claimed that cancer results from impaired mitochondrial metabolism. The increased glycolysis is thought to be a response to the hypoxic conditions characterising the microenvironment of malignant cells [6]. An upregulation of glycolysis is associated with a marked increase in glucose consumption, which can be observed by tumour imaging techniques such as positron-emission tomography. The conversion of pyruvate to lactic acid leads to microenvironmental acidosis and facilitates both invasion and metastasis [7,8]. In addition, lactic acid suppresses the proliferation of and cytokine production by human cytotoxic T lymphocytes and causes a significant decrease in their cytotoxic activity [9]. The latter finding may explain the frequently observed inability of the immune system to control aggressive cancer despite a specific T-cell response against tumour-associated antigens.

Therapies designed to target the anaerobic metabolism of tumours may preferentially kill malignant cells exhibiting this metabolic alteration. Promising experimental results in the treatment of certain types of tumours have been obtained with inhibitors of glycolysis [6] or of the pentose phosphate pathway [10], and with ketogenic diets [11]. Most malignant tumours are largely dependent on glucose for their growth and survival, but they are unable to metabolise ketone bodies for energy production [11]. A ketogenic diet restricts the glucose supply while providing the body with adequate energy substrates in the form of fat for generating ketone bodies. In 1995 Nebeling and coworkers described the long-term management of paediatric astrocytoma patients by a ketogenic diet. In addition to its beneficial effect on tumour growth, the diet improved the patients' nutritional status [12].

Ketogenic diets aim to induce ketosis, a physiological response of the body to limited dietary carbohydrate intake with consequent exhaustion of the glycogen content in liver and skeletal muscle resulting in the body's use of fat for energy. During ketosis, the liver starts to degrade fatty acids and to form acetyl-CoA in fatty acid oxidation. Acetyl-CoA can then be diverted into the ketone bodies acetoacetate and β-hydroxybutyrate (β-OHB), the major ketone body in plasma [13]. Ketone bodies are transported from the liver to other tissues where they can be reconverted to acetyl-CoA. Although glucose is the preferred fuel, ketone bodies can supply 2% to 6% of the body's energy needs after an overnight fast and 30% to 40% after a 3-day fast. The brain can also utilise ketone bodies to supply up to 60% of its metabolic energy needs [13].

When applied under caloric restriction, different ketogenic diets supplemented with either lard or soybean oil have been shown to have an inhibitory effect on tumour growth [14-16]. However, under caloric restriction the diets led to a dramatic weight loss in contrast to *ad libitum *feeding. Several groups have focused on the impact of lipid oils on both tumour growth and body weight. Without caloric restriction, an anti-tumour effect was demonstrated for diets rich in omega-3 fatty acids and medium chain (6–12 carbons) triglycerides (MCT), which are not present in lard and scare in soybean oil [17-20]. Different researchers have repeatedly shown in animal experiments that the growth of human cancer xenografts can be slowed by omega-3 fatty acid-enriched diets [21-23]. Still, the potency of omega-3 fatty acids to reduce the risk of cancer in humans remains controversial [24-27]. An anti-angiogenic effect of omega-3 fatty acids has been demonstrated *in vitro *and *in vivo *[28-30]. A recent study revealed that the risk of prostate cancer can be modulated by the dietary omega-6/omega-3 polyunsaturated fatty acids ratio in prostate-specific Pten knockout mice [31]. Since Pten acts as a suppressor of Akt signalling, which itself is intimately linked to the glycolytic phenotype, these experiments provide a link between lipid and glucose metabolism in pathological conditions.

To determine the impact of a ketogenic diet on tumours exhibiting aerobic glycolysis, we compared the effects on growth and survival of a nutritionally balanced carbohydrate-restricted diet supplemented with a mixture of vegetable oils and oil extracts possessing elevated levels of polyunsaturated omega-3 fatty acid and MCT with those of a standard diet in a mouse xenograft model. We report a tumour-suppressive effect of this diet with respect to growth, necrosis, and neovascularization of subcutaneously implanted tumours in nude mice, feeding *ad libitum*.

## Methods

### Animals

Twenty-four nude mice (6 to 8-week-old females) of the NMRI strain obtained from Harlan Winkelmann GmbH (Borchen, Germany) were maintained in groups of six animals per cage in laminar flow hoods in a pathogen-free environment. They were allowed access to food and water *ad libitum*. The study was reviewed and approved by the Animal Care Committee of the local government in accordance with the national guidelines for animal care (German Law for the Protection of Animals).

### Tumour cells of the human cell line 23132/87

We used cells of the human gastric adenocarcinoma cell line 23132/87 [32], which were kindly provided by Prof. H.P. Vollmers, Institute of Pathology, University of Würzburg. The cell line is available from the German Collection of Microorganisms and Cell Cultures (Deutsche Sammlung von Mikroorganismen und Zellkulturen GmbH (DSMS), Braunschweig, Germany). The tumour cells were cultured as a monolayer in RPMI 1640 medium supplemented with 100 U/ml penicillin, 100 μg/ml streptomycin, 2 mmol/L glutamine, 1 mmol/L sodium pyruvate, 10% heat-inactivated fetal calf serum (all products from Invitrogen-GIBCO, Germany). The cells were routinely tested for mycoplasma contamination to ensure that only negative cells were used [33].

### *In vitro *characterisation of cell line 23132/87 tumour cells

Cells of the carcinoma cell line 23132/87 were tested *in vitro *for their ability to metabolise glucose to lactate in the presence of oxygen. This aerobic glycolytic activity of glucose metabolism may correlate with a unique tumour phenotype characterised by a higher metastatic and invasive potential [6]. The glucose uptake by the tumour cells was monitored with the fluorescent deoxyglucose analog 2-(N-(7-nitrobenz-2-oxa-1,3-diazol-4-yl)amino)-2-deoxyglucose (2-NBDG) [34]. Since no corresponding benign gastric cancer cell line was available, HUVEC cells (Promocell, Heidelberg, Germany) were used as control cells and tested in parallel. In brief, cells were seeded at 100,000/0.5 ml/well in a 24-well plate and after 24 h of culture the cells were washed with PBS and cultured in glucose-free DMEM medium (PAA Laboratories, Linz, Austria) for 15 min. The cells were then incubated with 0.01, 0.1 and 1 mmol/l 2-NBDG (Invitrogen/Molecular Probes, Karlsruhe, Germany) for 10, 30, and 60 min. at 37°C. The 2-NBDG uptake was stopped by washing the cells twice with ice-cold PBS. Negative controls were cells incubated with 2-NBDG on ice and cells incubated without 2-NBDG at 37°C. Following trypsination, cells were resuspended in 100 μl PBS/10% FCS and counterstained with 1 μg/ml Propidium Iodide (PI). Ten thousand PI-negative cells were measured in a FACS Scan flow cytometer (Becton Dickinson, Heidelberg, Germany) and data were analysed by the free WinMDI 2.8 software package (The Scripps Research Institute, USA). Lactate production was assessed in cell supernatants of 20,000 cells/100 μl/well in a 96-well plate incubated for 24 h with the L-lactic acid detection kit (Roche/R-Biopharm, Darmstadt, Germany) following the manufacturer's instructions. The colour reaction was measured in an absorption plate reader (Sunrise Absorbance Reader; Tecan, Crailsheim, Germany) at 340 nm. For this purpose the measuring volume was scaled down to 200 μl.

### *In vivo *growth of cell line 23132/87 tumour cells

For the *in vivo *experiments a freshly thawed tumour cell aliquot was cultured for up to three passages *in vitro *not more than 3 weeks prior to injection into nude mice. All mice received tumour cells from the same cell passage. The cultured cells (nearly 70–80% confluence) were harvested with trypsin EDTA (PAA) and the viability of the detached cells was routinely checked with the trypan blue exclusion test. All tumour cells prepared for inoculation were highly viable (>90%). They were inoculated subcutaneously in both hind flanks (2.5 × 10^6 ^cells per flank). Tumour nodules appeared approximately 8–10 days following cell injection and the larger of the two was selected for analyses. All data presented is based on these tumour nodules. No animal died from tumour growth. The tumour size was measured with calipers and the tumour volume V_T _(mm^3^) was calculated using the ellipsoid formula *A*^2 ^× *B *× *π/6*, where A represents the smaller diameter. Endpoint for the experiments was attainment of a tumour volume between 600 and 700 mm^3 ^(target tumour volume), with the interval between subcutaneous tumour cell inoculation and the endpoint defined as the survival time. Tumours reaching the target tumour volume were dissected and the final target volume and wet weight were determined. Subsequently, they were cut through the median, one part was fixed in formalin and embedded in paraffin, the other part was embedded in Tissue-Tek (Sakure Finetek Europe B. V., Zoeterwoude, The Netherlands) and snap frozen in liquid nitrogen.

### Diets and feeding

All mice received a nutritionally balanced diet (altromin 1430) provided by the special animal feed manufacturer Altromin GmbH & Co. KG, Lage, Germany, prior to the inoculation of tumour cells. This standard diet was supplied in pellets delivering 12.8 kJ/g gross energy and consisting of 7.0% fat, 23.8% protein, and 36.4% carbohydrates (Table 1). The ketogenic diet consists of a mixture of fresh, high quality food homogenized into a paste using a standard food processor. A similar diet is being examined in an ongoing clinical trial at the University of Würzburg Hospital for the treatment of incurable tumour patients. The paste consists of 40.7% curd cheese (40% fat), 19.9% mackerel, 8.1% blue veined cheese, 8.1% white veined cheese, 8.1% bacon, 4.0% Tavarlin bread (Tavartis, Otzberg, Germany), 8.1% flaxseed, and 2.7% sesame seed. The paste (737 g) enriched with 300 ml of Tavarlin oil and 100 ml Tavarlin lactate drink (both Tavartis) was autoclaved, aliquoted in petri dishes under sterile conditions and stored at -20°C. Three dishes per six animals were thawed overnight at +4°C prior to feeding. This diet delivers 15.4 kJ/g gross energy and consists of 35.5% fat, 13.0% protein, and 0.2% carbohydrates (Table 1) and contains 21.45% MCT. The Tavarlin oil mixture consists of 28.7% saturated fatty acids, 35.7% unsaturated fatty acids, and 35.6% polyunsaturated fatty acids with a ratio of omega-6/omega-3 of 1.77:1. Chemical analyses of the nutrient contents were performed by the Chemical Laboratory Hameln (Dr. Kaiser & Dr. Woldmann GmbH, Hameln, Germany). Following tumour cell injection on day 0 the animals (n = 24) were randomly split into two equal feeding groups: standard diet (SD) and ketogenic diet (KD). Tumour size and body weight of all animals were measured every second/third day.

**Table 1:** Composition of the standard (SD) and ketogenic diets (KD) used in this study.

**Component **^1)^	**SD **^2)^	**KD **^3)^
**Fat**	7.0	35.5 ^4)^
**Carbohydrate**	36.4	0.2
**Protein**	23.8	13.0
**Fiber**	17.3	14.8
**Energy (kJ/g)**	12.8	15.4
**Ketogenic ratio **^5)^	0.1 : 1	2.7 : 1

^1) ^Data as gram per 100 g diet.

^2) ^The SD diet contains 9.0 g water and 6.5 g ashes.

^3) ^The KD diet contains 34.4 g water and 2.1 g ashes.

^4) ^The fat source consists of a mixture of vegetable oils from flaxseed and hempseed with elevated levels of omega-3 fatty acid and MCT (see Methods).

^5) ^Calculated according to the following formula: Fats/(Protein + Carbohydrates).

### Measurement of plasma glucose and beta-hydroxybutyrate (β-OHB)

Blood glucose and β-OHB levels were measured on the day of tumour cell injection (day 0) and every week thereafter until the last day of the experiments before tumour resection. Measurements were done with a blood glucose and ketone monitoring system (Precision Xtra, Abbott Laboratories, Abbott Park, Illinois, U.S.A.) and corresponding test strips (Abbott GmbH & Co. KG, Wiesbaden, Germany) using 2 μl of peripheral blood collected from a snipped tail vein of each animal.

### Immunohistochemistry

Tumour tissue sections 2-μm thick were deparaffinized with xylene and rinsed in decreasing concentrations of ethanol prior to unmasking by heating for 5 min with 10 mmol/L sodium citrate buffer in a microwave oven at 600 W. After irrigating in distilled H_2_O, the endogenous peroxidase was quenched with 3% hydrogen peroxide in methanol for 10 min. Cryosections (5 μm) were fixed in acetone and subsequently air-dried. All sections were then washed with PBS, blocked for 15 min in 1% goat serum, and incubated with primary monoclonal mouse antibodies for 60 min. The following antibodies, diluted in a commercial antibody diluent (DAKO, Hamburg, Germany), were used: mouse-anti-transketolase like enzyme 1 (TKTL1; clone JFC12T10, Linaris GmbH, Wertheim, Germany), final dilution 1:400; mouse anti-pan cytokeratin (clone KL1, Immunotech, Marseille, France), final dilution 1:100; anti-Ki-67 antigen (clone MIB-1, DAKO), final dilution 1:50; and rabbit anti-glucose transporter type 1 (Glut-1; G3900-01, US Biologicals, Swampscott, MA, USA), final dilution 1:100. The slides were washed in PBS, incubated with biotinylated anti-mouse and anti-rabbit immunoglobulins (LASB-kit, DAKO), and treated with streptavidin-peroxidase (LASB-kit, DAKO) according to the manufacturer's protocol. After development in 5% 3,3'-diaminobenzidine (DAKO) and counterstaining with haematoxilin, the sections were dehydrated in graded ethanol and embedded in Vitro Clud (Langenbrinck, Emmendingen, Germany).

### Microvessel analysis

Cryosections of dissected tumours were stained with a rat anti-mouse CD34 antibody (RAM34, BD Pharmingen, Heidelberg, Germany), final dilution 1:100. Microvessel density was qualitatively assessed by examining the entire vital cellular zone of the tumours with a light microscope at 100× magnification.

### Determination of the size of necrosis in tumours of the KD and SD group

Sections corresponding to the median line were stained with haematoxyline-eosine and photographed at a magnification of 4× with a digital camera. Images of each whole section were imported into Microsoft PowerPoint and all areas with morphologically well-defined necrosis were circled using the free-hand drawing function of the program at high magnification. The complete area of necrosis per section was quantified using the "analyse particles" option of the public domain Java image processing program ImageJ 1.34 s (downloaded from the National Institutes of Health (NIH), Bethesda, MD, USA) and expressed as percentage of the section's total area.

### Statistical analysis

GraphPad Prism 4.0 software (Statcon, Witzenhausen, Germany) was used for statistical analyses. Body weight, tumour growth, plasma glucose, blood ketone levels and necrotic areas were analysed by Mann-Whitney U test to show significant differences between the KD and SD groups after the nonparametric rank order test of Puri and Sen [35]. Probability values below 0.05 were considered significant.

## Results

### Characterisation of cells of the human gastric adenocarcinoma cell line 23132/87 *in vitro *and *in vivo*

**Figure 1:**
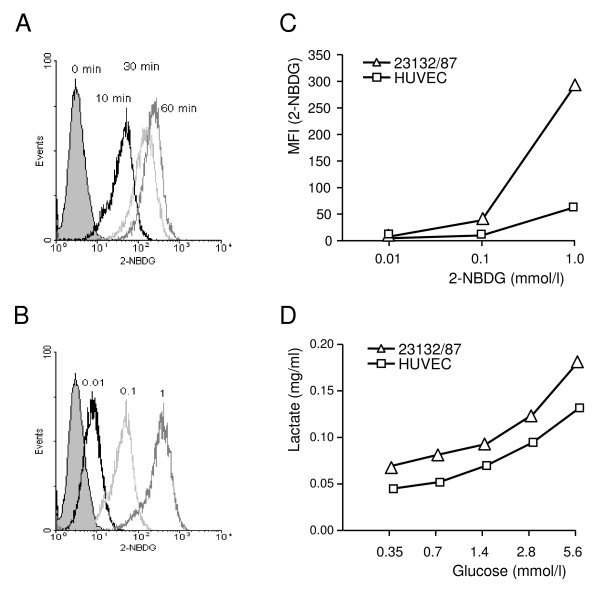
**Glucose consumption and lactate production by tumour cells of the gastric carcinoma cell line 23132/87**. (A) Time-dependent glucose uptake. The glucose uptake was measured with the fluorescent deoxyglucose analog 2-NBDG by flow cytometry. Tumour cells were incubated with 0.1 mmol/l 2-NBDG for 10, 30, and 60 min under normoxic conditions. The non-filled curves indicate the proportion of cells incorporating 2-NBDG and the filled curve represents the background staining of cells incubated with 2-NBDG on ice. (B) Concentration-dependent glucose uptake. Tumour cells were incubated with 0.01, 0.1, and 1 mmol/l 2-NBDG for 10 min. The filled curve represents cells incubated without 2-NBDG. (C) The 2-NBDG uptake of gastric carcinoma cells in comparison with HUVEC. The cells were incubated for 10 min with 0.01, 0.1, and 1 mmol/l, respectively. The flow cytometric data represents the total tumour cell population minus dead cells. MFI: Mean fluorescence intensity; ΔMFI = (MFI_2-NBDG_)-(MFI_unstained cells_). (D) Lactate production. Lactate concentration in the culture medium was measured as described in Methods. Lactate production by tumour cells and HUVEC depends on glucose concentration in the culture medium but shows an increase in gastric cancer cells. Data in A-D are from one of three independent experiments.

Cells of the carcinoma cell line 23132/87 were tested *in vitro *for their ability to metabolise glucose to lactate in the presence of oxygen. First, the glucose consumption was measured with 2-NBDG glucose by flow cytometry. The 2-NBDG glucose uptake, which is time and dose-dependent (Fig. 1A,B), was higher in gastric carcinoma cells than in HUVEC (Fig. 1C). Second, supernatant from overnight cultures with 2 × 10^5 ^tumour cells/ml in the presence of 5.6 mmol/l glucose was found to have 0.18 mg/ml L-lactate, whereas the same number of HUVEC produced 0.13 mg/ml L-lactate. In addition, the lactate production depends on glucose concentration in the culture medium (Fig. 1D). In addition, all tumour sections of both the KD and SD groups showed a clear surface expression of Glut-1 and cytoplasmic expression of TKTL1 (Additional File 1).

### Course of body weights

**Figure 2:**
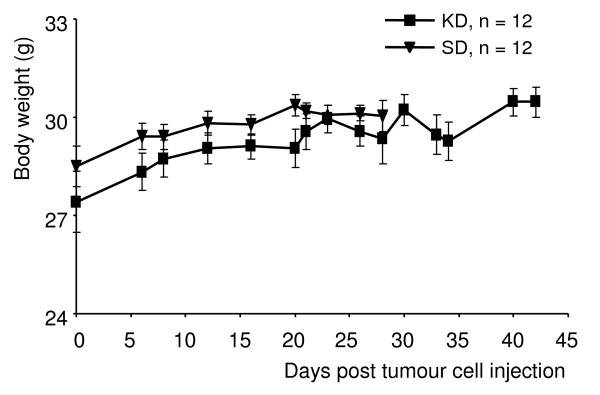
**Changes in the body weight of tumour-bearing nude mice on the ketogenic and standard diets (n = 12 mice per group)**. Following tumour cell injection on day 0, animals of the KD group were fed the unrestricted ketogenic diet, animals of the SD group continued with the standard diet. Values are expressed as mean ± standard deviation. The slopes of the mean body weights of KD and SD animals are not significantly different (P = 0.065).

All animals of the KD group readily accepted the unrestricted ketogenic diet and showed a steady increase in body weight over a period of up to 45 days (Fig. 2). The mean body weights of the KD and SD animals prior to tumour cell injection were 27.4 ± 3.3 g vs. 28.5 ± 2.2 g (difference not significant, P = 0.62), respectively, and at experiment's end: 29.6 ± 2.0 g vs. 29.9 ± 1.3 g (difference not significant, P = 0.18).

### Animal survival

**Figure 3:**
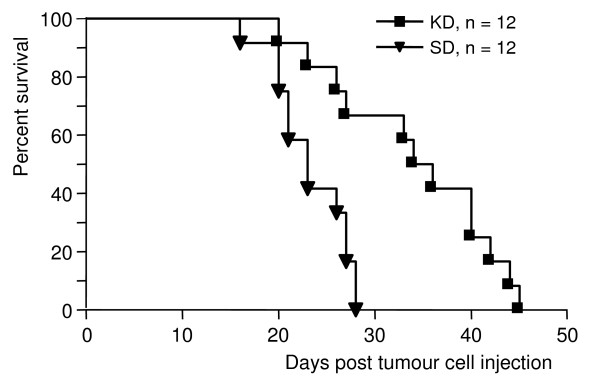
**Influence of the ketogenic diet on animal survival times**. Data are expressed as Kaplan-Meier survival curves (n = 12 mice per group). Survival in the KD group was significantly prolonged compared to that in the SD group (P = 0.001).

The last two animals in the SD group reached the endpoint on day 28 after tumour cell injection (Fig. 3). At this time point, only 4 of 12 animals in the KD group reached the target tumour volume. The last animal of the KD group reached the endpoint on day 45. The mean survival time of animals in the KD group was 34.2 ± 8.5 days, in the SD group 23.3 ± 3.9 days. Overall, application of the unrestricted ketogenic diet was highly significantly associated with survival (P = 0.0054, Fig. 3).

### Analyses of tumour growth

**Figure 4:**
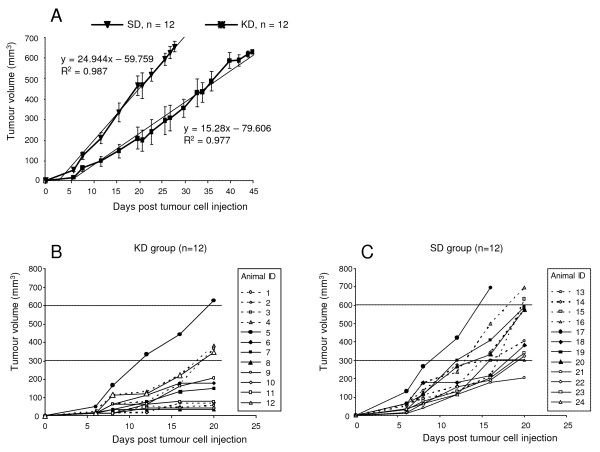
**Influence of the ketogenic diet on tumour growth**. (A) Shown is the mean tumour volume ± standard deviation as well as the respective regression lines of KD and SD animals. Endpoint for the experiments was attainment of a tumour volume between 600 and 700 mm^3^. Slopes are significantly different (P = 0.021). R^2 ^(coefficient of determination). (B-C) Shown are tumour volumes for the individual animals of the KD and SD groups for the first 20 days after tumour cell inoculation.

All 24 nude mice receiving a subcutaneous injection of tumour cells of the gastric adenocarcinoma cell line 23132/87 showed growth of solid tumours. On the day of tumour cell injection, animals of the KD group were switched from the standard diet to the ketogenic diet. Tumour progression was followed by measuring tumour volume over time. The difference between the mean tumour volume curves of KD and SD animals is significant (P = 0.021, Fig. 4A). The slopes of the regression lines for the mean tumour volumes in the KD and SD group are 15.28 versus 24.94. Comparison of the tumour growth curves for individual animals of the KD and SD groups reveals a strong delay in tumour growth in KD animals compared to SD animals during the first 20 days after cell inoculation (Fig. 4B, 4C). At day 20, 8 of 12 KD animals had tumours with a volume below 300 mm^3^, whereas this was true of only 2 of 12 SD animals. As described in Methods, tumour growth to more than 600 mm^3 ^(but below 700 mm^3^) determined the end of the experiment and the interval between tumour cell injection and endpoint was defined as the survival time. At the experiments' endpoint, neither tumour volumes (658 ± 32.6 mm^3 ^KD vs. 662 ± 35.1 mm^3^, P = 0.88) nor tumour weights (397 ± 45.6 mg KD vs. 413 ± 32.8 mg, P = 0.75) differed significantly between the two groups. The results indicate that the determination of the target tumour volume (between 600–700 mm^3^) is an accurately definable endpoint of experiment.

### The ketogenic diet influenced plasma β-OHB levels but not glucose levels

Mice of the KD group achieved ketosis within six days after feeding was started and their β-OHB levels remained continuously higher (between 1.2- and 2.4-fold) than those of the SD animals. The β-OHB levels of KD and SD animals differed highly significantly after day 6 (P < 0.001, Table 2). In contrast, no significant difference was noted between the blood glucose levels of the KD and SD groups. These findings are consistent with studies showing that a ketogenic diet does not lower plasma glucose levels when given *ad libitum *[16]. At the end of experiment the blood glucose levels were 5.5 ± 2.7 mmol/l KD vs. 6.3 ± 1.3 mmol/l SD (P = 0.59) and the β-OHB levels were 1.3 ± 0.5 mmol/l KD vs. 0.6 ± 0.1 mmol/l SD (P < 0.001). The serum insulin levels, measured with ELISA (ultrasensitive mouse insulin ELISA from Mercodia, Sweden, following the manufacturer's instructions) at the end of experiment, were slightly reduced in KD animals in comparison to SD animals (63.3 ± 32.5 pmol/l vs. 72.0 ± 41.0 pmol/l), but the difference was not significant (P = 0.84).

**Table 2:** Serum levels (mmol/L) of β-OHB in tumour-bearing animals of the KD and SD groups on different days after tumour cell injection (day 0).

**KD**	**Days after tumour cell inoculation**					
	
	0	6	8	20	23	28
Mean	0.5	0.9 *	1.2 *	1.6 *	1.3 *	1.2 *
± SD	0.1	0.2	0.2	0.6	0.4	0.3
						

**SD**	**Days after tumour cell inoculation**					
	
	0	6	8	20	23	28

Mean	0.5	0.5	0.5	0.6	0.7	0.6
± SD	0.09	0.09	0.07	0.06	0.08	0.06

KD animals achieved ketosis within 6 days after feeding the ketogenic diet (Table 1) and remained significantly higher than those of SD animals (* P < 0.001). In contrast, serum glucose levels were not significantly different between SD and KD animals (see Results).

### Histological and immunohistological analysis of human tumours derived from the 23132/87 cell line

**Figure 5:**
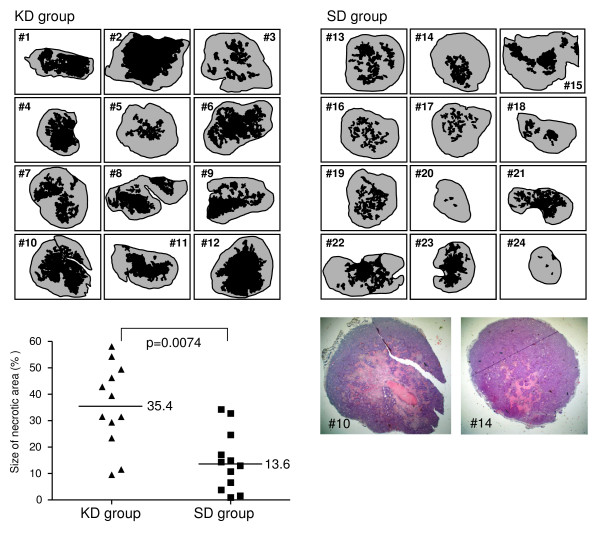
**Size of the necrotic areas (in black) in tumours of animals of the KD (animals 1–12) and SD (animals 13–24) groups**. The total area of necrosis per section was quantified and expressed as the percentage to the total area of the section as described in Methods. Tumours of the KD group had significantly larger areas of necrosis than tumours of the SD group (P = 0.0074). A representative H&E histology for KD and SD animals is shown.

Histology of tumour nodules revealed striking differences in the size of necrotic areas of the two groups (Fig. 5). Tumours from animals of the KD group exhibited highly significantly larger necrotic areas (35.4% of total area in median) than tumours of the SD group (13.6% in median, P = 0.0074). Concerning the vital areas of the tumours, the two groups did not differ appreciably in expression levels of Glut-1, TKTL1, and the proliferation marker Ki-67 antigen (Additional File 1). All cells, which were nearly 100% Ki-67 positive, expressed Glut-1 and were clearly positive for TKTL1. Analysis of the CD34-stained tumour sections revealed that the size of the areas with vital tumour cells correlated with the density of vessels spreading from the subderma into the tumour nodule. Interestingly, tumours of the KD group appear to have had fewer vessels than tumours of the SD group (Additional File 2).

## Discussion

This study was designed to test whether a ketogenic diet can inhibit the growth of tumours of the human gastric adenocarcinoma cell line 23132/87 in a xenograft model. The tumour cells demonstrated increased glucose consumption and lactate production *in vitro*. They were positive for TKTL1, a marker enzyme of aerobic glycolysis [36], whose expression has been shown to correlate with a poor prognosis in a variety of carcinomas [37-40]. The ketogenic diet used here provides average protein and is low in carbohydrates and high in fat enriched with omega-3 fatty acids and MCT. Compared to the applied standard diet, the unrestricted ketogenic diet had a retarding effect on tumour growth and resulted in larger necrotic areas within the tumours. Blood glucose levels in the KD group were unaltered, but its ketone body levels were significantly elevated compared to those of the SD group. Since our study does not allow to decide whether the effects of the diet are due primarily to omega-3 fatty acids and MCT, or to a combination, further studies are needed to address this issue.

The observation that unrestricted access to the ketogenic diet retarded tumour growth contrasts with data on another ketogenic diet, KetoCal, a commercially available diet for children with epilepsy [16]. The therapeutic effect of KetoCal on tumour growth was apparent in adult mice only when their caloric intake was restricted, which resulted in a 20% – 23% loss in body weight within eight days after start of feeding. KetoCal provided to animals *ad libitum *produced no marked loss in body weight but also had no influence on tumour growth [16]. In contrast to the calorically restricted KetoCal diet, we observed neither significant weight loss nor reduced blood glucose levels in our animals, although the tumour suppressive effects of the diets were comparable. Our data therefore suggest that an effective metabolic tumour therapy is not necessarily accompanied by reduced blood glucose levels. A possible cause of the observed delaying effect of the ketogenic diet on tumour growth is the high levels of omega-3 fatty acids and MCT in the diet. An antitumour effect has been demonstrated for both omega-3 fatty acids and MCT in patients and experimental models [17-21,24].

Cancer patients with advanced incurable cancer are typically threatened by cancer cachexia, characterised by progressive weight loss, mainly due to loss of fat and skeletal muscle, and anorexia [41]. Although cancer cachexia accounts for about 20% of cancer deaths, its underlying mechanisms are not known in detail [42]. To improve the quality of life and survival time of incurable patients, it is important to avert the onset of cachexia. Calorically restricted diets are therefore not suitable as treatment for these patients. Ketogenic diets, however, with high fat, adequate protein and low carbohydrates, have been shown to prevent or limit the protein catabolism in skeletal muscle [43]. In 1995 Nebeling et al. proposed a ketogenic diet rich in MCT as a successful therapeutic option in pediatric cancer patients [12]. Barber et al. later reported that the combination of fish oil and an energy-dense nutritional supplement increased body weight in cachectic cancer patients [44]. A non-restricted ketogenic diet may thus indeed be capable of benefiting cachectic cancer patients when supplemented with adequate lipids. The ketogenic diet described in this study induced both a slight increase in body weight and a slower growth rate of human tumour cells in nude mice.

Tumours of the KD group were characterised by significantly larger necrotic areas than those of the SD group. This finding may be explained by the restricted glucose supply in the KD group. However, we did not find significant differences in blood glucose levels of KD and SD animals. This observation indicates that glucose is synthesised from noncarbohydrate precursors by a process called gluconeogenesis. Fearon et al. even found higher blood glucose levels in ketotic, tumour bearing rats than in ketotic, non-tumour bearing rats. The authors considered that the inability of the ketogenic diet to reduce tumour growth was due to persistently high glucose levels [45]. In contrast, different feeding studies with carbohydrate-free diets showed significantly lower levels of circulating glucose compared to carbohydrate-enriched diets [46]. Nebeling et al. described that within 7 days of initiating ketogenic diet, blood glucose levels declined to low levels [12]. In addition, the authors calculated from results of PET scans a 21.8% average decrease in glucose uptake at the tumour site. One possible explanation for the significantly delayed tumour growth despite constant blood glucose levels in mice of the KD group is the ability of ketogenic diets to significantly reduce blood insulin levels [47]. It is widely accepted that frequently elevated levels of insulin can stimulate tumour growth [48]. We found slightly reduced insulin levels in KD animals, but the difference to insulin levels of SD animals was not significant.

Another possible explanation for the antitumour effect of the ketogenic diet is its ability to delay tumour take. Following tumour cell injection the animals of the KD group were fed with the ketogenic diet. The comparison of the individual tumour volumes of KD animals reveals that the unrestricted ketogenic diet delayed tumour growth strongly in the first 20 days after tumour cell inoculation. Five tumours in the KD group did not grow, 3 tumours grew slightly, whereas only 4 tumours grew as fast as the tumours of the SD group. The observation that the ketogenic diet delays the tumour cell take could be clinically significant for prevention of metastatic tumour cell take. However, further studies are required to accurately discriminate the effects of the ketogenic diet.

The significantly larger necrotic areas in the centre of tumours grown in the KD mice correlate well with the reduced microvessel density in these tumours. The suppression of neovascularization may be provoked by the anti-angiogenic effect of omega-3 fatty acids [28-30], as well as by reduced levels of lactate/pyruvate in glucose-starved tumour cells, which are able to stimulate angiogenesis via HIF-1-mediated transactivation of VEGF [49]. Suppressed neovascularisation may further inhibit an adequate supply of glucose to the centre of the tumours. In aggressive tumour cells such a severe limitation of substrate produces a state termed 'metabolic catastrophe', which enhances necrosis. The therapeutic induction of metabolic catastrophe was recently proposed as an approach to killing "unkillable" tumour cells [50]. Since glucose-fermenting tumour cells have been shown to have substantially enhanced resistance to several anticancer drugs [51,52], the combined application of conventional chemotherapy and metabolic tumour therapy may represent an effective approach for targeting both fermentative and respiratory cell populations.

Transplantation of human cancer cells or tumour biopsies into immunodeficient mice is a commonly used xenograft model [53]. Since this model precludes T cell-mediated cellular immunity, the results do not reflect any immunosuppressive effects of lactate as a by-product of glucose fermentation. Due to lactate's suppressive effect on the cellular immune response [9], it is conceivable that the ketogenic diet we applied would have more profound effects in a model that allows a T-cell response directed against tumour-associated antigens. Additional studies using other models are necessary to further explore the true potential of metabolic tumour therapy in a functionally active immune system.

## Conclusion

In this pilot study we demonstrate that a carbohydrate-restricted diet supplemented by lipids rich in omega-3 fatty acids and MCT delays the growth of glucose fermenting tumours. The effect did not depend on caloric restriction and there was no loss of body weight. Further studies are needed to address the impact of metabolic therapy on additional tumour-relevant functions such as invasive growth and metastasis. The ketogenic diet described here may provide a promising strategy for targeting glucose fermenting cell populations *in vivo*.

## Abbreviations

ATP: adenosine triphosphate; β-OHB: beta-hydroxybutyrate; CK: cytokeratin; CoA: coenzyme A; DMEM: Dulbecco's modified Eagle's medium; EDTA: ethylenediamine tetraacetic aicd; FCS: fetal calf serum; Glut-1: glucose transporter type 1; H&E: haematoxyline-eosine;HUVEC: Human Umbilical Vein Endothelial Cell; 2-NBDG: 2-(N-(7-nitrobenz-2-oxa-1,3-diazol-4-yl)amino)-2-deoxy-D-glucose; PBS: Phosphate-buffered saline; 23132/87: human gastric adenocarcinoma cell line; PET: positron-emmission tomography; RPMI 1640 medium: Roswell Park Memorial Institute; TKTL1: transketolase like enzyme 1.

## Competing interests

JFC and RW declare a potential conflict of interest due to the possible application of Tavarlin oil for therapeutic purposes and TKTL1 for therapeutic and diagnostic purposes.

## Authors' contributions

CO drafted the manuscript, designed the study, set up the experiments, participated in data collection, analysed and interpreted the results and provided images and figures. UK drafted the manuscript, carried out the histological analysis and analysed the results, and provided images and figures. BI contributed to the conception of the study and acquired funding from the Interdisciplinary Centre for Clinical Research (IZKF) of the University of Würzburg. BM participated in the feeding of the mice, tumour cell injection and data analysis. NP performed the measurements of glucose uptake and lactate production and participated in data analysis. HUV carried out the histological analysis and participated in data interpretation. RW revised the article for intellectual content. AT participated in editorial support and research funding. JFC participated in the study design, experimental concept, and data interpretation. He was responsible for production of the ketogenic diet. All authors read and approved the final manuscript.

## Pre-publication history

The pre-publication history for this paper can be accessed here:



## Supplementary Material

Additional file 1 **Immunohistochemical analysis of representative tumours of the KD (animal 9) and SD (animal 16) groups**. The carcinoma cells (pan cytokeratin-positive) located within the viable zone around the necrosis have proliferated (Ki-67 antigen-positive) and exhibit a glycolytic phenotype (TKTL1-positive). The expression of Glut-1 correlated with the strong glucose uptake and lactate production shown in Fig. 1 (Magnification: ×250). Click here for file

Additional file 2 **Influence of the ketogenic diet on vascularity in tumours of the human gastric adenocarcinoma cell line 23132/87**. Representative tumour sections from the KD (animal 11) and SD (animal 13) groups are shown. Vessels were stained with the rat anti-mouse CD34 antibody RAM34 as described in Methods (Magnification: ×400). Click here for file
