# Supplementary figures and images for: Growth of human gastric cancer cells in nude mice is delayed by a ketogenic diet supplemented with omega-3 fatty acids and medium-chain triglycerides
Source: BMC Cancer. 2008 Apr 30;8:122. doi: 10.1186/1471-2407-8-122 (PMC2408928; doi:10.1186/1471-2407-8-122)

KD group (#9)

SD group (#16)

CK

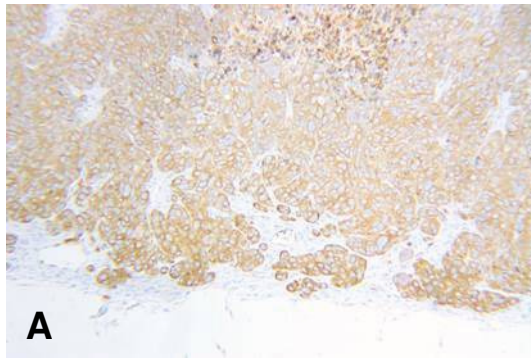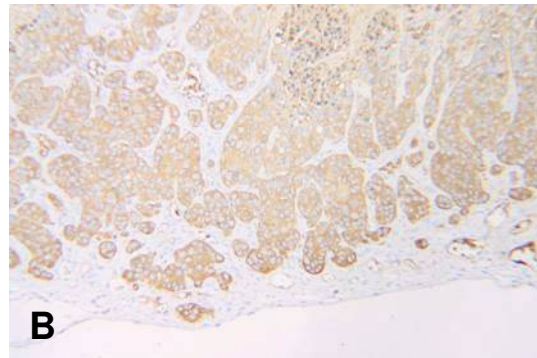

Ki-67

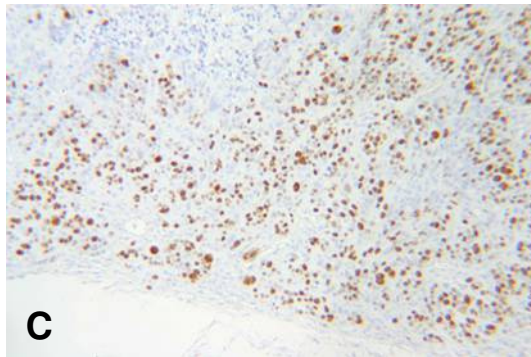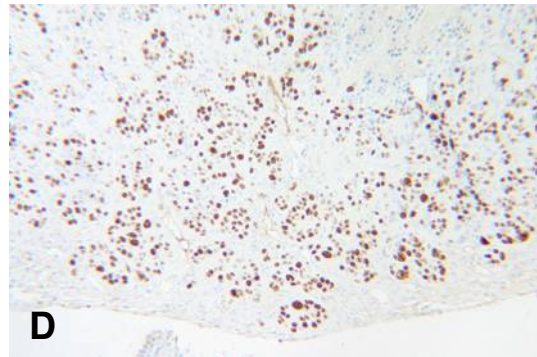

Glut-1

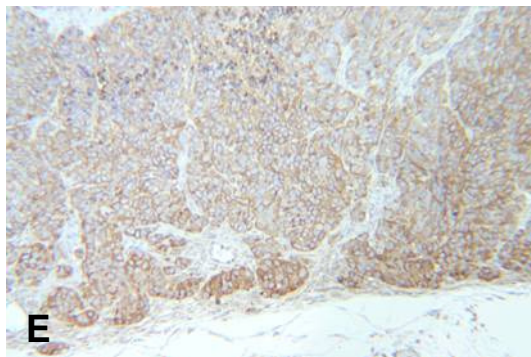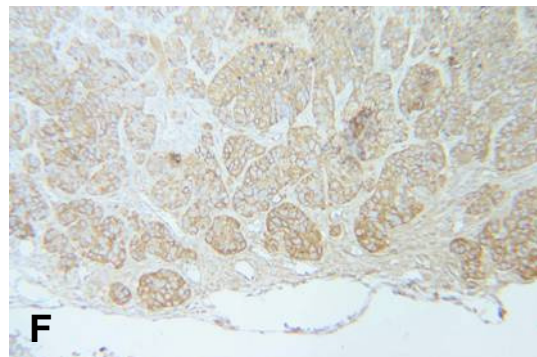

TKTL1

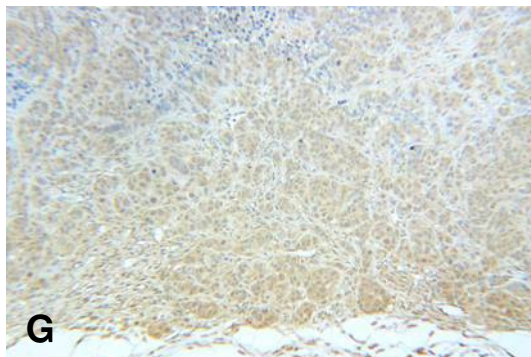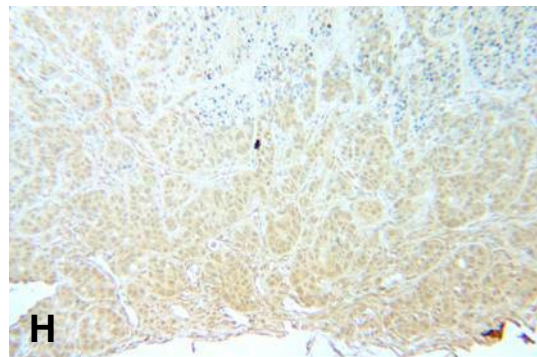

Supplement: Additional file 1 — Immunohistochemical analysis of representative tumours of the KD (animal 9) and SD (animal 16) groups. The carcinoma cells (pan cytokeratin-positive) located within the viable zone around the necrosis have proliferated (Ki-67 antigen-positive) and exhibit a glycolytic phenotype (TKTL1-positive). The expression of Glut-1 correlated with the strong glucose uptake and lactate production shown in Fig. 1 (Magnification: ×250). [file 1471-2407-8-122-S1.pdf]

**# 11**

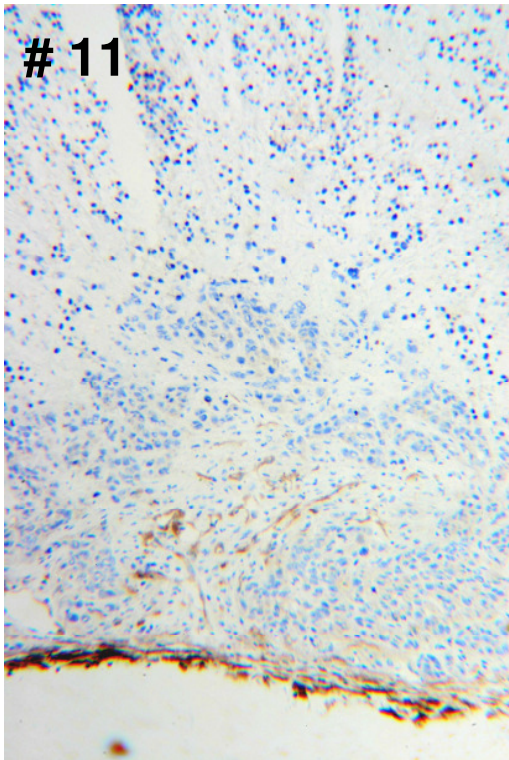

**#13**

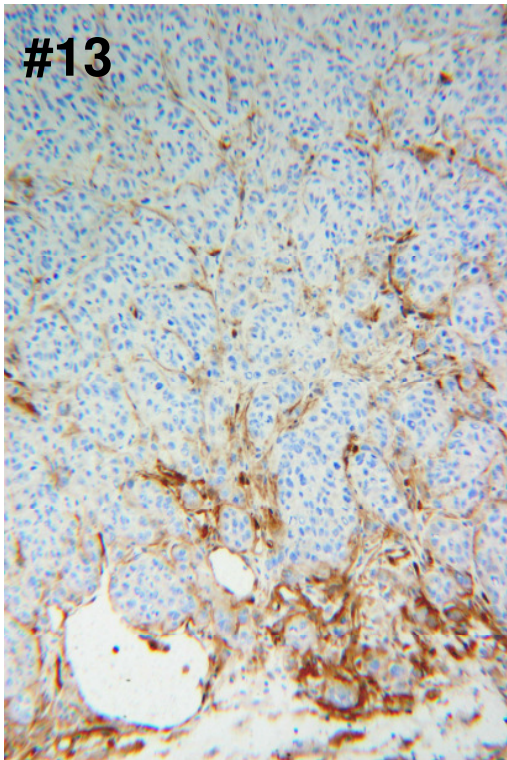

Supplement: Additional file 2 — Influence of the ketogenic diet on vascularity in tumours of the human gastric adenocarcinoma cell line 23132/87. Representative tumour sections from the KD (animal 11) and SD (animal 13) groups are shown. Vessels were stained with the rat anti-mouse CD34 antibody RAM34 as described in Methods (Magnification: ×400). [file 1471-2407-8-122-S2.pdf]
